# The SPORTS Participation Framework: illuminating the pathway for people with disability to enter into, participate in, and excel at sport

**DOI:** 10.1016/j.bjpt.2024.101081

**Published:** 2024-05-22

**Authors:** Georgina Leigh Clutterbuck, Ricardo Rodrigues de Sousa Junior, Hércules Ribeiro Leite, Leanne Marie Johnston

**Affiliations:** a School of Health and Rehabilitation Sciences, The University of Queensland, Brisbane 4072, Australia; b Children's Motor Control Research Collaboration, The University of Queensland, Brisbane 4072, Australia; c Graduate program in Rehabilitation Sciences, Physical Therapy Department, School of Physical Education, Physical Therapy and Occupational Therapy, Universidade Federal de Minas Gerais, Brazil

**Keywords:** Disability, Paralympic, Parasport, Participation, Physical activity, Sport

## Abstract

**Background:**

Sports participation for people with disabilities exists at the intersection of health, sport, and education sectors. However, no common framework and language exist to describe the stages of sports participation.

**Objective:**

To present the background to the SPORTS Participation Framework, and how it can be used to illuminate the path that people with disability may travel to enter into, participate in, and enjoy and excel at all levels of sport.

**Method:**

The SPORTS Participation Framework includes six stages drawn from mainstream sports pathways and models used to classify barriers to sports participation for people with disabilities: (S) Screening, goal setting and individual preparation, (P) Practitioner led, peer-group sports interventions, (O) Organised junior entry-point sports programs, (R) Recreational sport (non-competitive), (T) Team competition (school/club representation), and (S) State, National, and International competition.

**Results:**

For each stage, this paper describes the content of sports activities, the context in which they are performed, key stakeholders, barriers to participation, available evidence, and case studies.

**Conclusions:**

The SPORTS Participation Framework presents a structure to navigate the stages of introducing and promoting lifelong sports participation for people with disabilities. It scaffolds clear communication, governance, and policy across health, sport, and education sectors, and supports clinicians and researchers to address barriers to participation at each stage to improve individual and population-wide participation in sport for people with disabilities.

## Highlights

**The SPORTS Participation Framework can be used across health, sport, and education sectors to**:

• Enhance interdisciplinary communication for children with disability with sports-focused goals.
• Identify and describe children's current stage of sports participation.
• Plan time- and cost-effective management strategies to reach goal stage of sports participation.

## Introduction

Sport provides individuals with enjoyable opportunities to develop meaningful relationships and improve and maintain physical and psychological wellbeing.^1^^,^^2^ It is particularly important that all people, including individuals with disabilities, have opportunities to participate in sport due to its importance in popular culture,^3^ health,^4^^,^^5^ education,^6^ and the economy.^7^ Unfortunately, people with disabilities participate in much less sport than their peers without disability.^8^^,^^9^ There is therefore considerable incentive to articulate the path that people with disability may follow to enter into, participate in, and excel at sport so that efforts can be made to support increased sports participation for this population at all stages.

Participation in sport is commonly seen either as an “entry point” (an intervention) or an “endpoint” (a goal).^10^ Entry point interventions aim to improve body structure and function impairments or activity limitations through participation in sports activities, for example, attending a soccer program (participation level intervention) to improve gross motor skills (activity level goal). In comparison, end point goals have sports participation as the ultimate aim, for example, a child may wish to participate in their school soccer team (participation level goal). Regardless of if sport is commenced as an entry or end-point activity, sports participation contributes to a positive, self-sustaining cycle whereby participation improves physical (e.g., fitness, gross motor function),^11^, ^12^, ^13^, ^14^, ^15^ psychological (e.g., exercise self-efficacy) ,^16^ and social (e.g., social integration and community *Participation*)^12^^,^^13^ wellbeing, which in turn increases attendance and enhances involvement in future participation.^10^^,^^17^, ^18^, ^19^

Sports participation for children and youth with disabilities exists at the intersection between the health, sport, and education sectors.^20^ Children with disabilities often spend their early lives closely linked to the health sector and must navigate the transition to the sport and education sectors if they are to access and sustain meaningful participation in sport. Research has highlighted the need for inclusive pathways that enhance partnerships between families, and health, sport, and education sectors to facilitate the complex transitions required for sustained participation.^21^^,^^22^ Being able to accurately identify and communicate individuals’ current and desired stage of participation is essential to support them to (1) set realistic and attainable end-point goals, and (2) identify and access the most effective entry-point interventions to reach these goals.

In the following sections, this paper will introduce the SPORTS Participation Framework, describe its theoretical background, define each stage of participation, and bring the SPORTS Participation Framework to life with fictional case examples.

## Introducing the SPORTS Participation Framework

The SPORTS Participation Framework (Supplementary material, Fig. 1) is an inter-sectorial framework that describes the six-stages that people with disability might progress through as they transition from receiving sports-focussed health interventions, to participating in wellbeing- and performance-focussed sports activities. Each stage is progressively more challenging in terms of the skills required to participate, the structure of the activities, and the level of competition involved. Where existing models may describe the different types of sports activities available for people with disabilities (e.g., the inclusion spectrum^23^), the SPORTS Participation Framework presents a functional pathway applicable regardless of type of sport. Moving through each stage provides opportunities for individuals to build skills during activities at the ‘just right challenge’ level,^24^ before transitioning to the next stage.

**Fig. 1:**
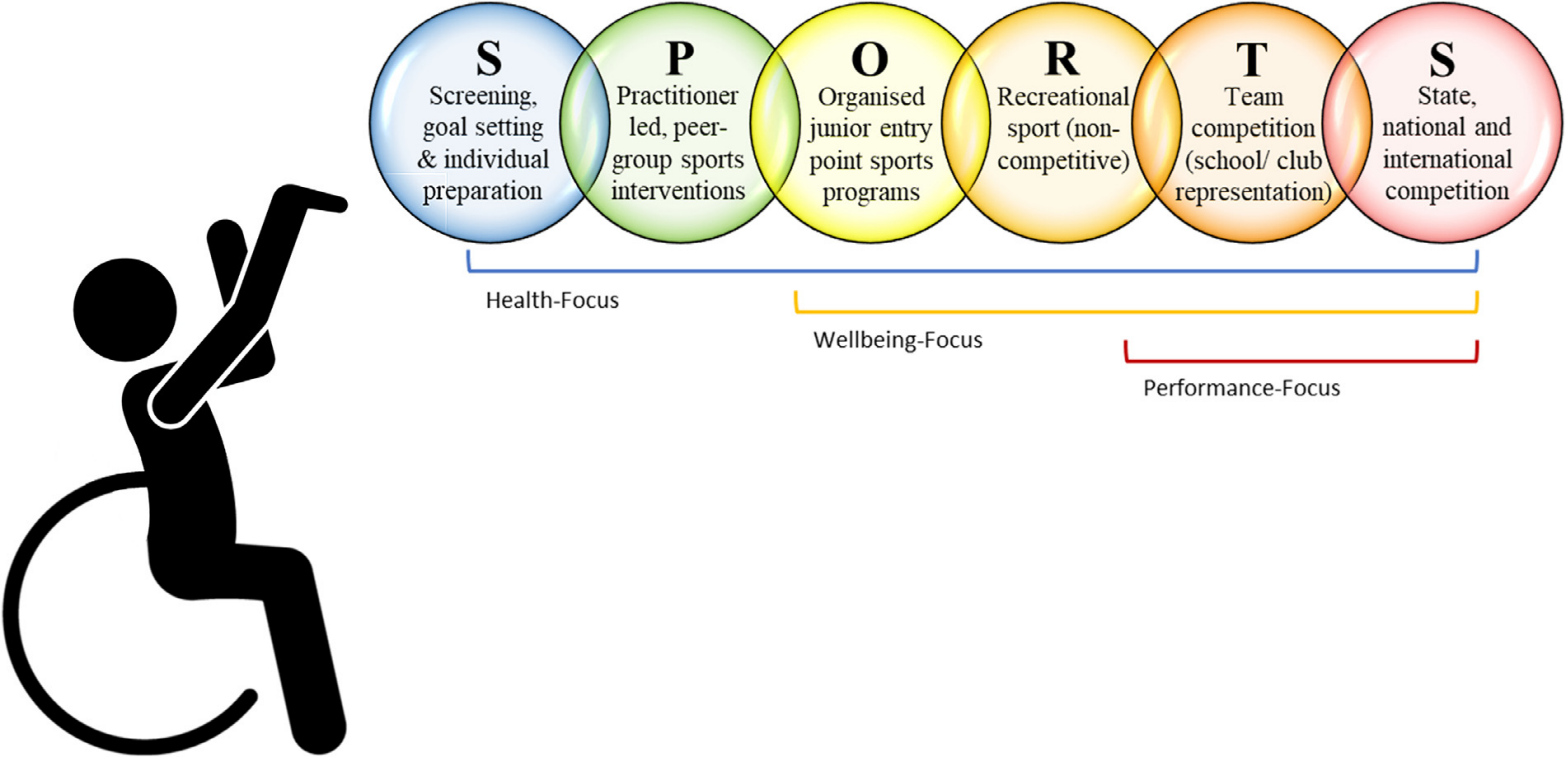
The SPORTS Participation Framework for people with disabilities. Adapted from.^25^ This work is licensed under a Creative Commons Attribution 4.0 International License https://creativecommons.org/licenses/by-nc-nd/3.0/au/deed.en; Fig. 1 Long description: A silhouette of a person in a wheelchair throwing a ball. The ball is depicted as a series of six circles with the letters S P O R T S to indicate the stage of sports participation. Each circle includes details of the stage name. (1) Screening, goal setting and individual preparation, (2) Practitioner led, peer-group sports interventions, (3) Organised junior entry-point sports programs, (4) recreational sport (non-competitive), (5) Team competition (school/ club representation), and (6) State, national and international competition. Brackets indicate that all SPORTS stages are health focussed, ORTS stages are wellbeing focussed and TS stages are performance focussed.

The SPORTS Participation Framework has been designed so that stakeholders across health, sport, and education contexts have a common language and a practical, integrated guide to conceptualise stages of sports participation, identify individual's current participation stage and future goals, and plan support strategies for life-long sports participation for people with disability.^25^^,^^26^ The stages have been conceptualised as a ball being hit, kicked, or thrown, with variations provided in the supplementary material to represent individuals with observable (e.g., amputation, use of mobility aid) or invisible (e.g., intellectual disability, autism) disabilities. The SPORTS Participation Framework is presented in terms of sports-focussed health interventions, wellbeing-focussed sports activities, and performance-focussed competitive sport to highlight the primary goals of participation at each stage. In this paper, each stage is described in detail, including the content of activities, the context in which they are performed, the key stakeholders involved, and an overview of available evidence.

## Theoretical background of the SPORTS Participation Framework

The SPORTS Participation Framework incorporates language and structures from key health, sport, and education frameworks to enhance intersectoral understanding for stakeholders (e.g., practitioners, coaches, physical educators, policy makers) and improve integration with current practice. Two key types of frameworks were integrated into the SPORTS Participation Framework; (1) frameworks used to classify the barriers and facilitators to participation, and (2) mainstream sports pathways. An overview of key frameworks, their relevence to sports participation for people with disabilty, and their integration into the SPORTS Participation Framework is provided below.

### Classification frameworks

Classification frameworks were drawn from health, sport, and education sectors and include the health-focussed ‘International Classification of Functioning, Disability and Health’ (ICF),^27^ the participation-focussed ‘family of Participation-Related Constructs’ (fPRC),^10^^,^^28^ and the physical activity-focussed ‘Physical Literacy’^19^ frameworks.

**Fig. 2:**
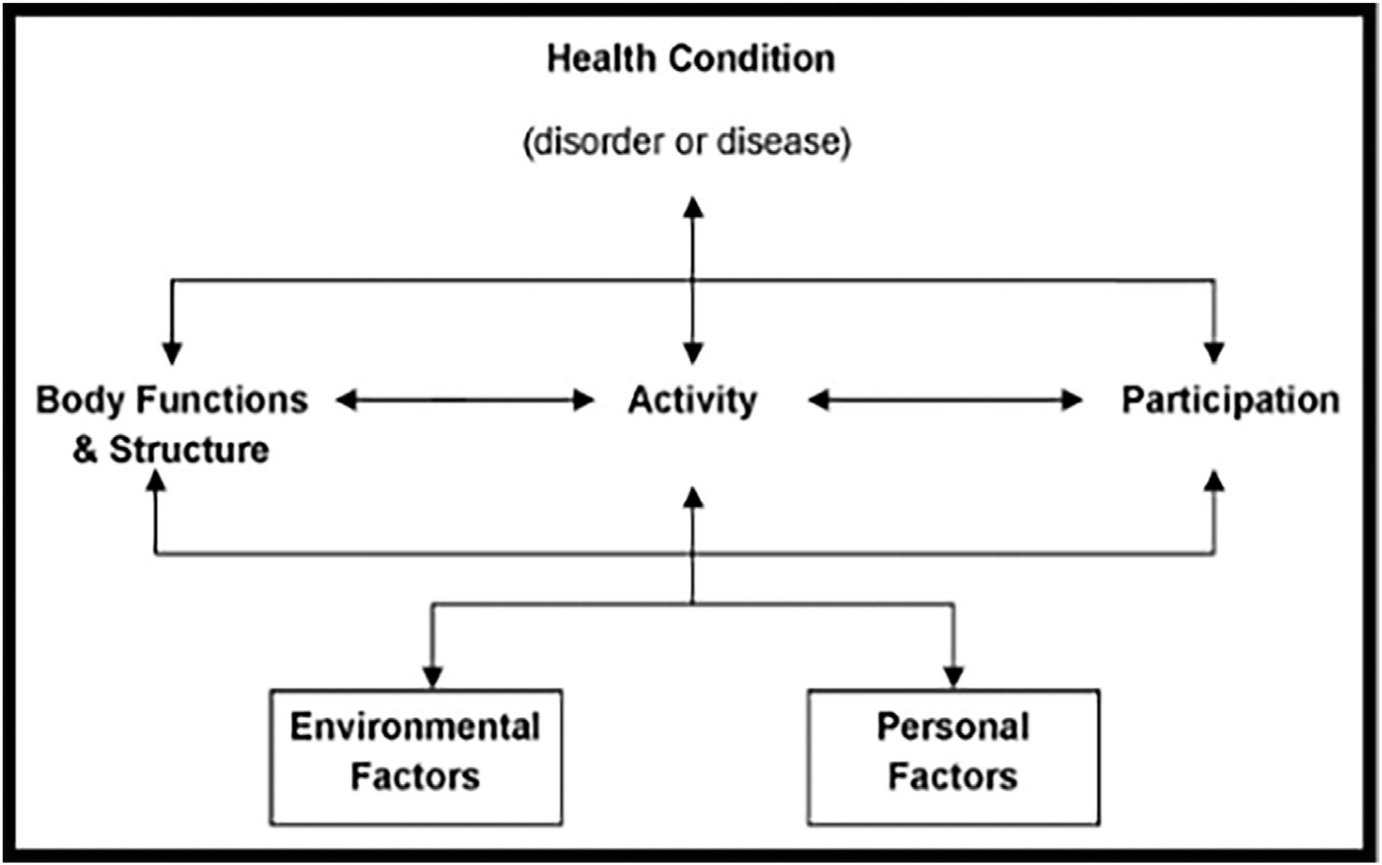
*The International Classification of Functioning, Disability and Health (ICF).*^27^*This work is licensed under a Creative Commons Attribution 4.0 International License*https://creativecommons.org/licenses/by-nc-nd/3.0/au/deed.en*;*Fig. 2*long description: A diagram with three levels. The top level includes one section ‘Health Condition (disorder or disease)’. The second level includes three sections: ‘Body Structure and Function’, ‘Activity’, and ‘Participation. The third level includes two sections: Environmental Factors and Personal Factors. There are bidirectional arrows between each item on a level, and with items on adjacent levels.*

The ICF (Fig. 2) is the primary framework used in health-focussed services globally.^27^ This person-centred, bio-psycho-social framework describes body structure and function impairments, activity limitations, and participation restrictions, within the context of the individual person and their environment.^29^ The ICF is a critical framework in the first ‘S’ stage of the SPORTS Participation Framework, where health professionals are typically responsible for screening for body structure and function impairments and activity limitations, and providing individual interventions with the ultimate aim of improving sports participation.^30^ The bi-directional relationships between body structures and functions, activity, and participation highlight the strong relationship between participation in sport, and improved health and wellbeing as individuals progress through SPORTS stages.

**Fig. 3:**
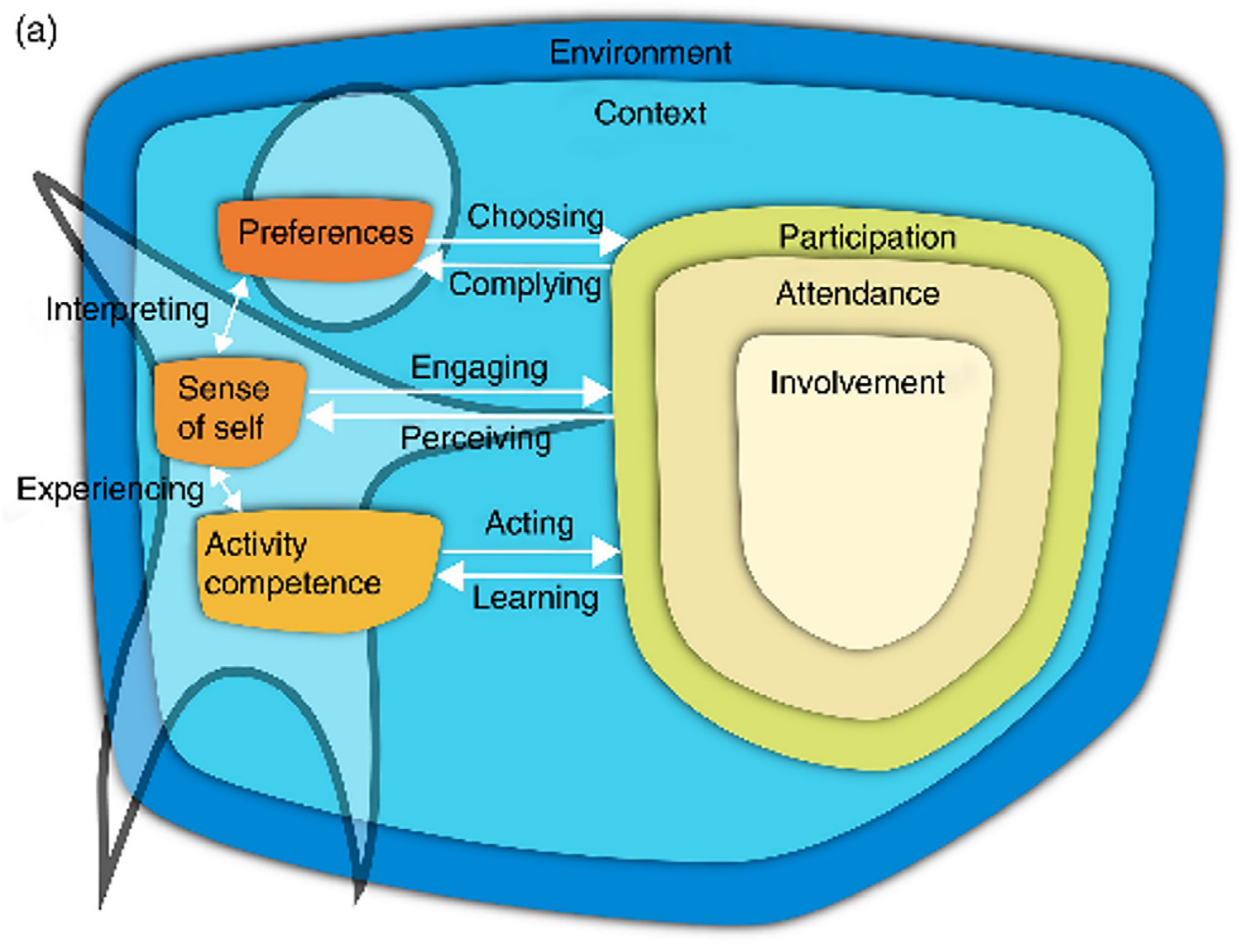

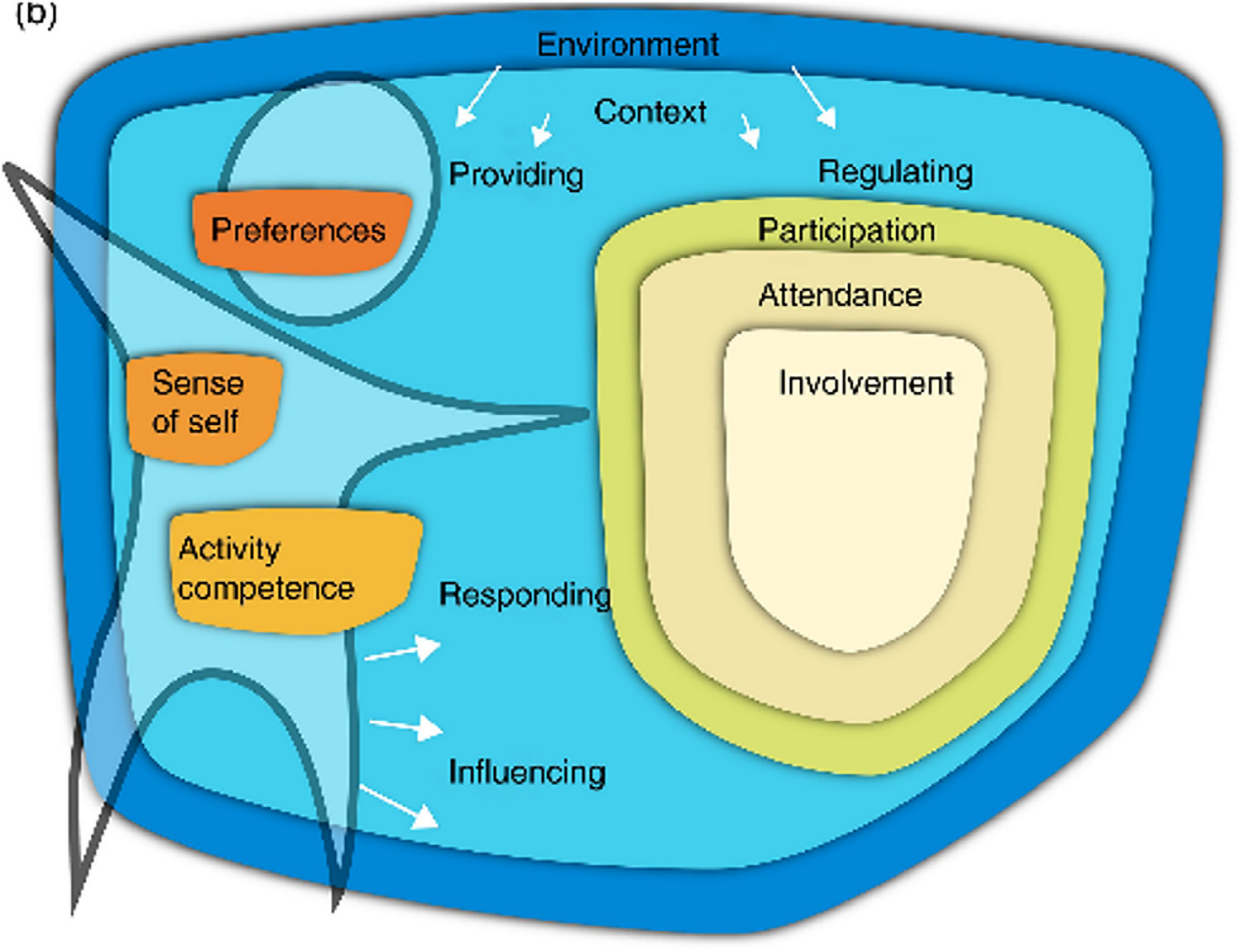
*a: Family of Participation-Related Constructs (fPRC) showing interactions with participation-related constructs.**^10^*; *This work is licensed under a Creative Commons Attribution 4.0 International License*https://creativecommons.org/licenses/by-nc-nd/3.0/au/deed.en*;*Fig. 3*a****Long description*:** A diagram shows a shape titled ‘Participation’, within which is a shape titled ‘Attendance’. Inside the ‘Attendance’ shape is a shape titled ‘Involvement’. Beside ‘Participation’ are shapes titled ‘Activity Competence’, ‘Sense of Self’ and ‘Preferences’ which are positioned on an outline of a person. All items sit within a border which is titled ‘Context’ which is within another border titled ‘Environment’ A bidirectional arrow between ‘Activity Competence’ and ‘Sense of Self’ is labelled ‘experiencing’. A bidirectional arrow between ‘Sense of Self’ and ‘Preferences’ is labelled ‘Interpreting’. The arrow from ‘Activity Competence’ to ‘Participation’ is labelled ‘Acting’. The reverse is labelled ‘Learning’. The arrow from ‘Sense of Self’ to ‘Participation’ is labelled ‘Engaging’. The reverse is labelled ‘Perceiving’. The arrow from ‘Preferences’ to ‘Participation’ is labelled ‘Choosing’. The reverse is labelled ‘Complying’. Fig. 3b: *Family of Participation-Related Constructs (fPRC) showing interactions with the environment and context.**^10^*; *This work is licensed under a Creative Commons Attribution 4.0 International License*https://creativecommons.org/licenses/by-nc-nd/3.0/au/deed.en*;*Fig. 3*b Long description***:** A diagram shows a shape titled ‘Participation’, within which is a shape titled ‘Attendance’. Inside the ‘Attendance’ shape is a shape titled ‘Involvement’. Beside ‘Participation’ are shapes titled ‘Activity Competence’, ‘Sense of Self’ and ‘Preferences’ which are positioned on an outline of a person. All items sit within a border which is titled ‘Context’ which is within another border titled ‘Environment’. The arrows from the outline of the person to the environment and context are labelled ‘Responding’ and ‘Influencing’. The arrows from the environment and context to the person are labelled ‘Providing’. The arrows from the environment and context to ‘Participation’ are labelled ‘Regulating’.

The fPRC (Fig. 3) expands on the ICF's *Participation* construct by describing the objective and subjective elements of participation (i.e., attendance and involvement) and participation-related constructs of (1) the individual and (2) the environment, including availability, accessibility, affordability, accommodability, and acceptability.^10^^,^^28^^,^^31^ The fPRC highlights transactions between participation and related constructs which are incorporated into the SPORTS Participation Framework in the iterative nature of attending a sports activity, developing sports skills, becoming more involved, and then transitioning up to the next SPORTS stage.

**Fig. 4:**
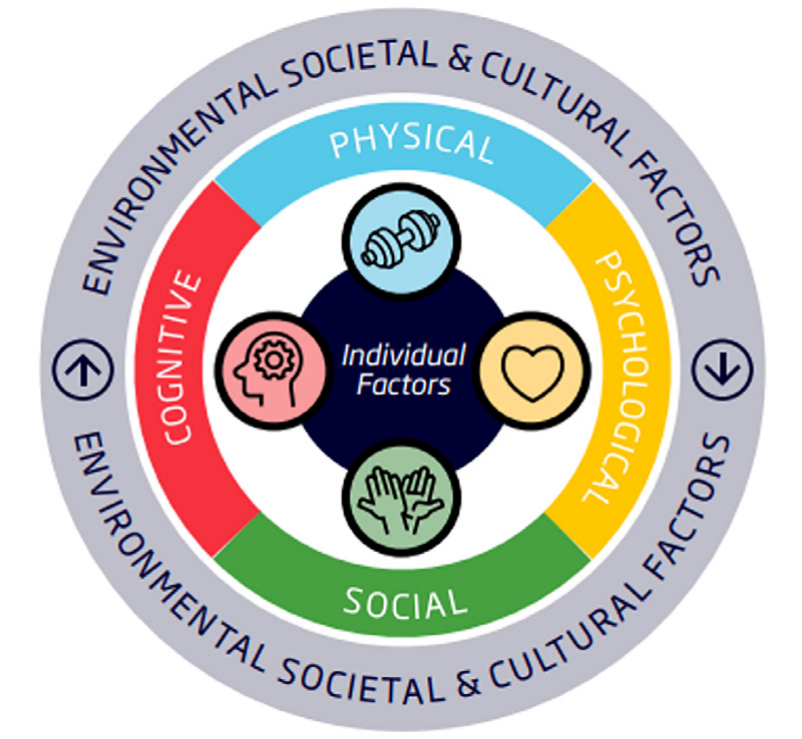
Australian Physical Literacy Framework^19^; This work is licensed under a Creative Commons Attribution 4.0 International License https://creativecommons.org/licenses/by-nc-nd/3.0/au/deed.en; Fig. 4 long description: A circle with the word ‘physical’ with an image of a hand weight, ‘cognitive’ with an image of a head with a cog where the brain would sit, ‘social’ with an image of two hands high fiving and ‘psychological’ with an image of a love heart in a circle around the words ‘individual factors’. This entire circle is surrounded by the words ‘environmental, societal and cultural factors’.

**Fig. 5:**
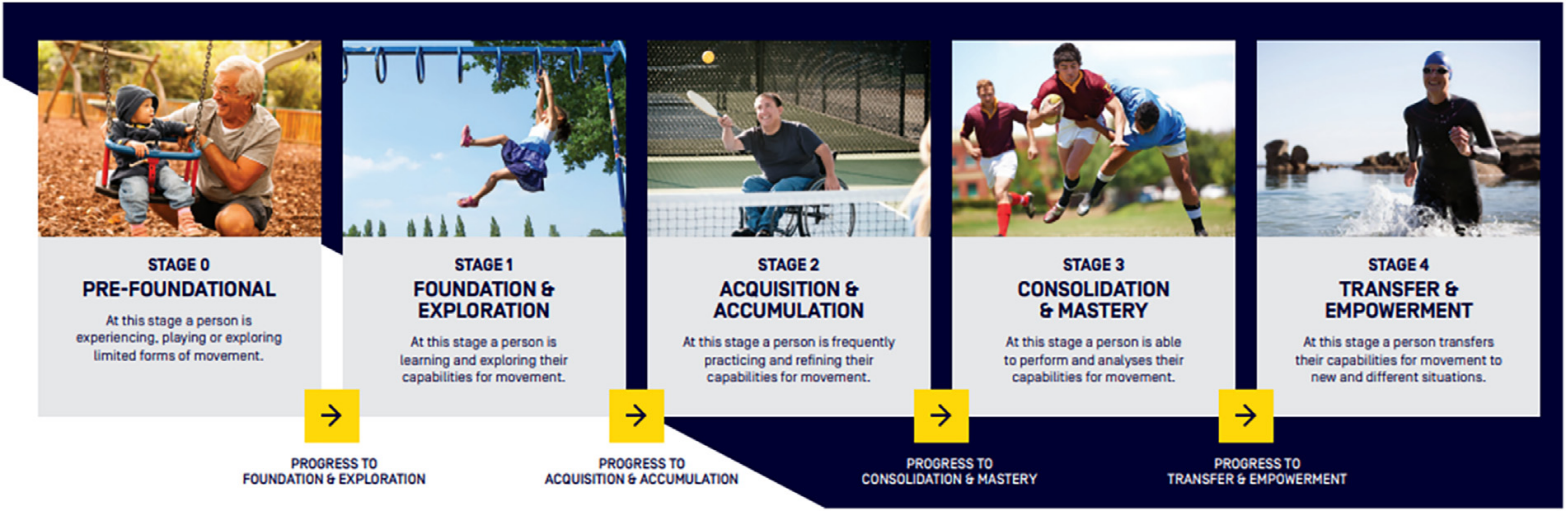
Australian Physical Literacy Framework Stages of Development^19^; This work is licensed under a Creative Commons Attribution 4.0 International License https://creativecommons.org/licenses/by-nc-nd/3.0/au/deed.en; Fig. 5 long description: Six boxes representing the six stages of development. Each box has a photo above the stage name and description. Stage 0: Pre-Foundational. Photo of a baby sitting in a swing at a park looking at an older man crouching next to them. Text reads ‘at this stage, a person is experiencing, playing or exploring limited forms of movement’. Arrow to stage 1: Foundation & Exploration. Photo of a young child swinging on monkey bars. Text reads ‘At this stage a person is learning and exploring their capabilities for movement’. Arrow to stage 2: Acquisition & accumulation. Photo of a middle-aged man in a wheelchair hitting a tennis ball over a net with his racquet. Text reads ‘at this stage a person is frequently practicing and refining their capabilities for movement’. Arrow to stage 3: Photo of a young man holding a football under their arm as they avoid being tackled. Text reads ‘consolidation & mastery. Text reads ‘at this stage a person is able to perform and analyse their capabilities for movement’. Arrow to stage 4: Transfer & empowerment. Photo of an adult in a wetsuit running through thigh-high water at the beach with a smile. Text reads ‘at this stage a person transfers their capabilities for movement to new and different situations’.

The Physical Literacy Framework (Fig. 4) is a physical-activity specific framework primarily used in sport and education sectors. The Australian Physical Literacy Framework provides a mechanism to describe the physical, social, cognitive, and psychological skills needed to participate in enjoyable physical activity across the lifespan,^19^^,^^32^^,^^33^ and can be used to describe the demands of each SPORTS stage. In particular, the *Stages of Development*^19^ (Fig. 5) is incorporated into the SPORTS Framework through the iterative pattern of increased sports participation, improved activity competence and transition to subsequent SPORTS stages.

While the ICF, fPRC, and Physical Literacy have been developed separately, there is significant overlap between them. Table 1 highlights this overlap, however it should be noted that while there are similarities between components of these three frameworks, this does not constitute exact overlap and each framework should be reviewed in detail prior to use.

**Table 1:** Alignment between the ICF, fPRC, and Physical Literacy models.^26^

ICF	Environmental Factors	Personal Factors	Body Structure/ Function	Activity		Participation	
fPRC	Environment & Context	Preferences	Participation-related constructs, i.e., Activity Capacity & sense of self			Participation Attendance	Participation Involvement
Physical Literacy	Environmental, Societal & Cultural Factors	Individual Factors	Physical	Social	Cognitive	Psychological	Participation in Physical Activity

### Sports pathways

The SPORTS Participation Framework was developed to align with international models of mainstream sports participation to ensure seamless integration into sports governance. Most national sports pathways emphasise elite-level performance-focussed sport (e.g., Sport England,^34^ UK Sport,^35^ Australian Institute of Sport^36^), despite the small proportion of participants (with or without disability) at this level. Sport Canada^37^ and Team USA^38^ present more balanced, population-based sports pathways which reflect that the majority of children will participate in recreational sport, or sport at the club or school level. The SPORTS Framework draws from this approach, balancing the fact that the majority of people participate in wellbeing-focussed (‘OR’ stages) and early performance-focussed (‘T’ stage) sport, while valuing the importance of representation in the highest levels of sporting competition (‘S’ stage).

## Stages of the SPORTS Participation Framework

### Sports-focussed health interventions: ‘SP’ stages

Sports-focussed health interventions are guided by health-focussed frameworks (i.e., the ICF and fPRC) and are implemented by health practitioners (e.g., physical therapists, occupational therapists, psychologists) to address sports-focussed goals. Most people with disabilities will receive health intervention in their early years, and people with sports-specific goals typically commence their sports pathway at this stage. While sports-focussed health interventions are provided to most people with disability with sporting goals, not all people with disability require health interventions to support their sports participation goals and some people without disabilities may benefit from receiving intervention at this stage.

The reason that most people with disabilities enter the SPORTS framework at the ‘SP’ stages is because they experience physical,^22^^,^^39^, ^40^, ^41^, ^42^, ^43^ cognitive,^44^ psychological,^22^^,^^41^^,^^43^ social,^45^ and environmental barriers to sports participation associated with their disability which impact their entry into later stages of the pathway. Interventions at the ‘SP’ stages should target any factors that hinder sports participation and should be family and person-centred, and provided by multidisciplinary teams which include relevant health practitioners such as physicians, physical therapists, occupational therapists, social workers, psychologists, physical educators, and speech and language pathologists.

#### Stage one: Screening, oal setting and individual preparation

The first ‘S’ stage (Fig. 1) includes three important components: Screening, goal setting, and individual preparation.

1. Screening

Comprehensive screening should identify an individual's current stage of SPORTS participation, and identify the barriers to attaining desired participation across ICF, fPRC, and physical literacy domains.

While there is strong evidence for the validity and reliability of standardised assessments of physical sport competence in people with disabilities, there is far less for the assessment of psychological, social, or cognitive impairments specific to physical activity participation in childhood,^46^, ^47^, ^48^ and even less for adults with disability. In the absence of standardised assessments, a multidisciplinary team should use comprehensive subjective examination, observation, and clinical reasoning to identify barriers to sports participation across all domains so that targeted intervention can be provided.

2. Goal Setting

The purpose of sports participation (i.e, entry or end point) should be established in the ‘S’ stage. Entry point goals may focus on body structure and function or activity level outcomes, e.g., to improve walking capacity, whereas end-point goals will focus on participation, e.g., attending weekly school soccer training. Participation goals should be set in accordance with the fPRC to include attendance at, and/or involvement in, activities at a specific SPORTS stage. There are a number of standardized goal attainment outcome measures (e.g., Goal Attainment Scale,^49^, ^50^, ^51^, ^52^ the Canadian Occupational Performance Measure^53^^,^^54^) with strong evidence for use with people with disabilities that can be used to support goal setting.

3. Individual preparation

Intervention at the ‘S’ stage should target the barriers to goal attainment identified. The individualised nature of this stage makes it difficult to efficiently report on the overall effectiveness of interventions. For example, strength training for a child for whom strength is not a barrier may improve strength but would be ineffective in improving participation. This is consistent with literature that reports that body structure and function interventions do not necessarily improve participation outcomes.^55^ Systematic reviews are available to support the effectiveness of specific types of intervention.^22^^,^^39^, ^40^, ^41^, ^42^, ^43^^,^^56^, ^57^, ^58^, ^59^, ^60^, ^61^, ^62^, ^63^, ^64^, ^65^, ^66^ However, it is essential that practitioners understand the mechanisms of action of interventions and use their clinical judgement to identify which interventions target specific barriers and will therefore be effective in supporting an individual's goal attainment.^67^

While one-off intervention may facilitate successful transition to later SPORTS stages, many people will continue to receive ‘S’ stage intervention throughout their life-long sports participation journey. As they transition up the stages, they will develop new goals, and may experience new barriers that require additional individual preparation.

#### Stage two: Practitioner-led, peer-group sports intervention

The ‘P’ stage (Fig. 1) acts as the bridge between traditional, individual health services in the ‘S’ stage, and the typical sports pathway presented by ‘ORTS’ stages.^25^, ^68^^,^^26^^,^^25^, ^69^, ^70^, ^71^ The ‘P’ stage is essential as it prevents people from becoming stuck in a cycle of health-focussed goals and interventions, and provides interventions that instead target transition into sports participation.

Practitioner-led, peer-group sports interventions target participation-related constructs of preferences, activity competence, and sense of self through the lens of physical literacy. The specific, transition-focus of ‘P’ stage interventions means that they act as a middle ground between individual health-focussed interventions in a clinical setting, and community sports activities which occur in large groups, in sports facilities, with sports coaches. ‘P’ stage interventions are short in length, conducted in community environments (e.g., sports courts) and provided in small groups. They expose people to different sports to facilitate the development of preferences, while continuing to reduce body structure and function impairments and activity level limitations identified in the first ‘S’ stage. In particular, the small-group context targets teamwork (social) and motivation (psychological), modified sports drills and games target sports-specific locomotor and object control skills (physical) and understanding of rules (cognitive), and the community environment targets enjoyment (psychological) and confidence (psychological).

Health practitioners are well suited to provide ‘P’ stage interventions due to their significant education and experience working with people with disabilities and their understanding of human movement. Interventions at the ‘P’ stage are designed for people who are not in need for individual healthcare services, but still need to be guided by a practitioner to develop their physical literacy skills prior to entering into sports-specific training with a sports coach. There is good evidence that practitioner-led, peer-group sports interventions improve participation in leisure-time physical activity for people with disabilities through the mechanism of improved physical literacy.^17^^,^^18^^,^^72^, ^73^, ^74^

### Wellbeing-Focussed sport: ‘OR’ stages

The ‘OR’ stages (Fig. 1) represent the start of most mainstream sports pathways.^26^^,^^34^^,^^36^, ^37^, ^38^ These stages are wellbeing-focussed in that they are non-competitive and champion participation in enjoyable physical activity with its associated health and wellbeing benefits. Wellbeing-focussed sports activities are conducted in community sports facilities in larger groups than ‘P’ stage interventions. The content and design of wellbeing-focussed programs reflects the culture in which they are provided. For example, in Australia, common sports include netball, cricket, soccer, and Australian football, whereas in Brazil, they are more likely to include handball, athletics, and basketball.^69^^,^^70^

Wellbeing-focussed sports programs are generally designed for the majority of people without disabilities. Mainstream programs are popular and therefore have good availability. However, as they are developed for people with age-appropriate sports activity competence*,* people with disabilities frequently experience additional barriers to participation. At an individual level, these barriers can be managed by sports coaches modifying activities, or additional individual support from a support worker or volunteer, and should be identified at the first ‘S’ stage. At a program level, sporting organisations should provide widespread coach education and training, minimum standards regarding physical accessibility of program sites, and improved availability of all-abilities, or disability specific programs such as AFL's StarKick program (Australian ‘O’ stage program) or Associação Mineira de Reabilitação's Sport-therapy program (Brazilian ‘R’ stage program)^75^ to specifically cater for people with disability who do not wish to, or are not able to participate in mainstream wellbeing-focussed programs.

#### Stage three: Organised junior entry-point programs

Organised junior entry-point programs are designed to provide modified sports activities to young children who are entering sport for the first time.^76^ They are provided in the community, by volunteer or paid sports coaches to support the transition to formal sport. ‘O’ stage programs simplify sports drills and games by modifying equipment (e.g., using larger balls) and rules (e.g., smaller team size, play area, or game length), however, compared to ‘P’ stage interventions, provide less support to individual children. While children with disabilities are typically welcome in ‘O’ stage programs, some children will require additional supports as identified in ‘S’ stage screening such as attending a program specifically developed for children with disabilities. The very small number of disability-specific programs can therefore be a significant barrier to participation.

#### Stage four: Recreational sport

Recreational sport is provided in the community by paid or volunteer sports coaches or with family, friends, or peers and provides opportunities to enhance wellbeing without the expectation of high-level performance.^77^^,^^78^ As recreational sport is not constrained by the strict rules that apply to competitive sport, there is greater flexibility to accommodate varying needs of people with disabilities by modifying components of the activity or environment. This flexibility is beneficial to people with disabilities who may be unable to perform a sports activity in the same way as their able-bodied peers, or whose abilities may fluctuate over time. Systems such as rotating players between positions or teams, not keeping score, and/or minimising spectators’ focus on winning mean that recreational sport may be preferred by individuals who do not want to, or are not able to play at a competitive level due to their personal preferences, focus on academics or employment, or their economic or family situation.

The absence of recreational sport for school-aged children is a significant barrier to continued sports participation, particularly for children with disabilities.^45^ Individuals who are unable to access recreational sport may experience decreased enjoyment when participating in competitive sport due to feelings of being unwelcome or excluded, and are more likely to drop out of sport as activities become less flexible.^22^^,^^79^ These children typically do not reengage in sports activities later in life. It is therefore essential that efforts are made to improve the availability of recreational sport for school-aged children with disabilities to reduce rates of drop-out and provide opportunities for them to maintain and enhance their health and wellbeing during adolescence and into adulthood.

### Performance-focussed competitive sport: ‘TS’ stages

Performance-focussed sport prioritises the optimisation of individual performance to best all other competitors. In addition, participation in activities at these stages will continue to have health and wellbeing outcomes. Activities at the ‘TS’ stages have systems for scoring individual bouts of competition (e.g., a single soccer game), and performance over time (e.g., a competition season). There is usually a tiered system of progression to a series final, where an overall winner is crowned. Performance-focussed sport is the primary focus of existing sports pathways and there are clear avenues to progress through school, club, and elite sporting competition for both able bodied, and para-athletes. National sporting associations oversee the pathway from school or club representation to elite competition for both streams of athletes.

Competitive para-sport uses standardised modifications to the activity, level of support, and classification to ensure that competition between athletes is fair. This allows para-sport to define success by the same standards of skill, fitness, power, endurance, tactical ability, and mental focus as mainstream sport.^80^ For example, the two-bounce rule in wheelchair tennis. Similarly, the use of human or technological support is allowed under strict, pre-defined rules e.g., sighted guides for blind athletes, or the use of a prosthesis, orthosis, or mobility aids for physically impaired athletes.

Competitive sport can hold benefits over recreational sport for those who can access and are interested in participating in competitive training programs. Training for performance focussed sports typically involves large volumes of physical activity, with consideration to balancing strength, endurance, aerobic, flexibility, and neuromotor components across a training cycle.^81^^,^^82^ There are established benefits of high-volume physical training for people with neurological impairments, and significant anecdotal evidence reported by Paralympic athletes.^81^^,^^82^

#### Stage five: Team competition

Team competition, where people compete in school or club teams, is the most common opportunity for school-aged children to participate in sport. This stage includes teams of athletes who compete in individual sports such as athletics or gymnastics. In the ‘T’ stage, competitive para-athletes often transition away from mainstream sporting competition and seek classification to compete against athletes of similar functional abilities.^83^ This may involve reengaging with health professionals to undertake the formal classification process. While the parasport pathway is well defined, there are significant barriers in terms of availability of ‘T’ stage programs for para-athletes as fewer participants exist in the same geographical location.^45^ This is particularly apparent in rural or remote areas, for athletes with high support needs, or in sports played in larger teams.

#### Stage six: State, national and international competition

State, national, and international competition is the most elite stage of performance-focussed sport. This includes world titles, and the Paralympic Games. While this final ‘S’ stage is still driven by sports coaches, health professionals once again become more heavily involved, this time to advise on optimal training to minimise disability-related impairments, enhance performance, and manage injury risk and rehabilitation.

Elite para-athletes have an important role in para-sport visibility. The opportunity to observe the elite sporting performance of people with disabilities provides real life examples for people with disabilities to aspire to. Where the Olympic games focus is wholly on sporting skill, the Paralympic committee also includes the call for para-athletes to “inspire and excite the world”.^84^ This includes enhancing the status of people with disability in society, as well as inspiring people with disability to participate in sport.^80^

Similar to at the Team competition stage, athletes in rural or remote areas, or with high support needs, may experience more environmental barriers to participating in State, National, or International competition. For example, in the 2024 Paralympic games in Paris, swimming events for participants at level S1 or S2 (affected by conditions such as complete quadriplegia) will not be offered.^85^ Athletes with high support needs may be discouraged from their competitive goals due to lack of event availability, or they may never be inspired to start participating due to reduced visibility of elite athletes with similar support needs to themselves.

## The SPORTS Participation Framework in practice

People with disabilities may not progress through all six SPORTS stages. Even though each SPORTS stage increases in complexity, structure, and competitiveness, people may skip stages, or cease progressing through stages. For example, a child who enters their SPORTS pathway at the ‘S’ stage of Screening, goal setting, and individual preparation, may transition directly to participation in ‘R’ phase recreational tennis with their family on the weekend. If this activity is sustainable, enjoyable, and meets the child and family's goals, there is no need to intervene to progress to performance-focussed sport participation, or to regress back to ‘O’ stage activities. The stages a person goes through, and the length of time and support they need at each stage, will depend on their preferences, activity competence, and the availability, accessibility, affordability, accommodability, and acceptability of programs in their area.

Case studies of how individuals may travel through the SPORTS Participation Framework are provided in the Appendix to facilitate a higher level understanding of the SPORTS Participation Framework in practice.

## Conclusion

The SPORTS Participation Framework presents the pathway from sports-focussed health interventions, into wellbeing-focussed sports activities, and finally into performance-focussed sports competition. The framework's six stages of increasing complexity, structure, and competitiveness are each governed by different sectors and stakeholders. This paper presents the SPORTS Participation Framework as a common language for sport, health, and education sectors, including researchers, health practitioners, sports coaches, and policy makers, and includes resources to use the SPORTS Participation Framework in future research and clinical practice. The SPORTS Participation Framework provides a scaffold to identify the barriers that people with disabilities experience to achieving their sports participation goals, and improve sports participation for this population through clinical practice, research, and governance.

## Declarations of competing interest

The authors have no conflicts of interest to declare.

## Appendix Supplementary materials

Image, application 1

Image, application 2
