# Supplementary material for: The SPORTS Participation Framework: illuminating the pathway for people with disability to enter into, participate in, and excel at sport
Source: Braz J Phys Ther. 2024 May 22;28(3):101081. doi: 10.1016/j.bjpt.2024.101081 (PMC11208908; doi:10.1016/j.bjpt.2024.101081)
Supplement: Supplementary file 1 [file mmc1.pdf]

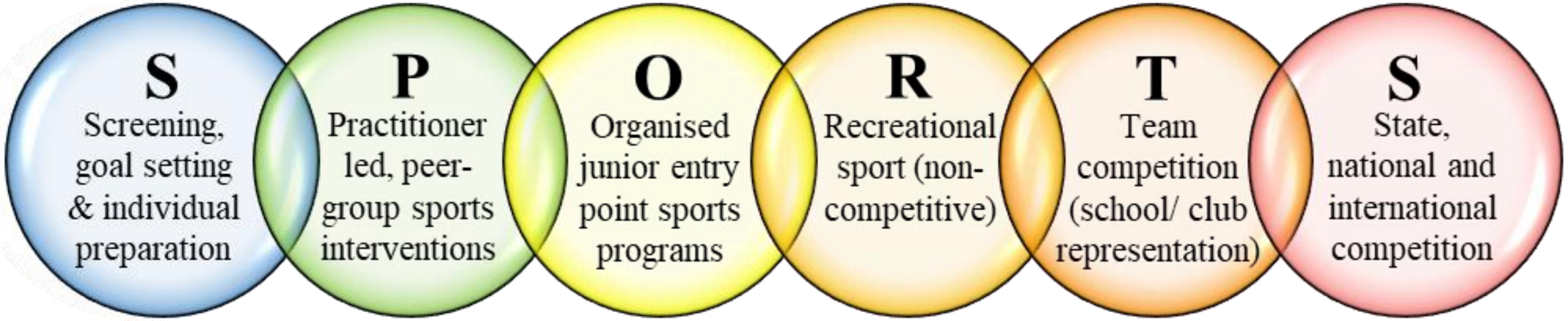

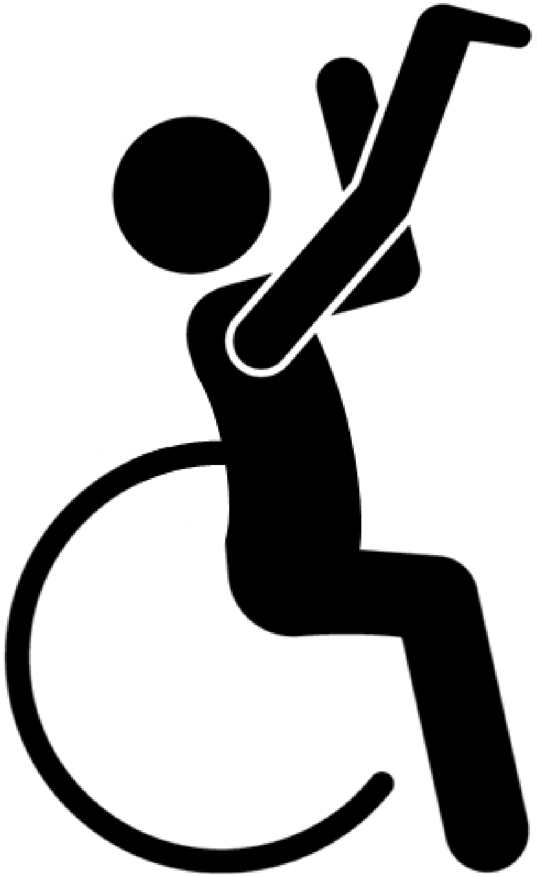

The SPORTS Participation Framework

for people with disabilities

Children's Motor Control
Research Collaboration

Clutterbuck, G. L., de Sousa Junior, R. R., Leite, H. R., & Johnston, L. M., (2024) *The SPORTS Participation Framework: Illuminating the pathway for people with disability to enter into, participate in, and excel at sport.* Brazilian Journal of Physical Therapy

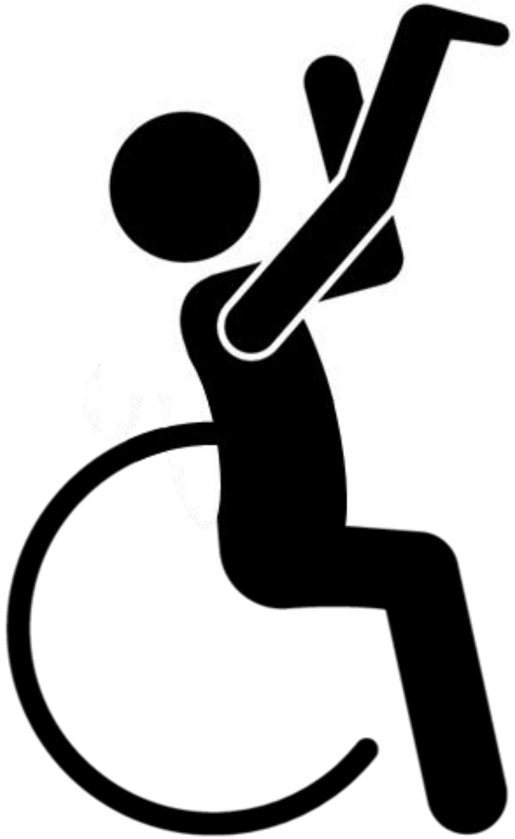

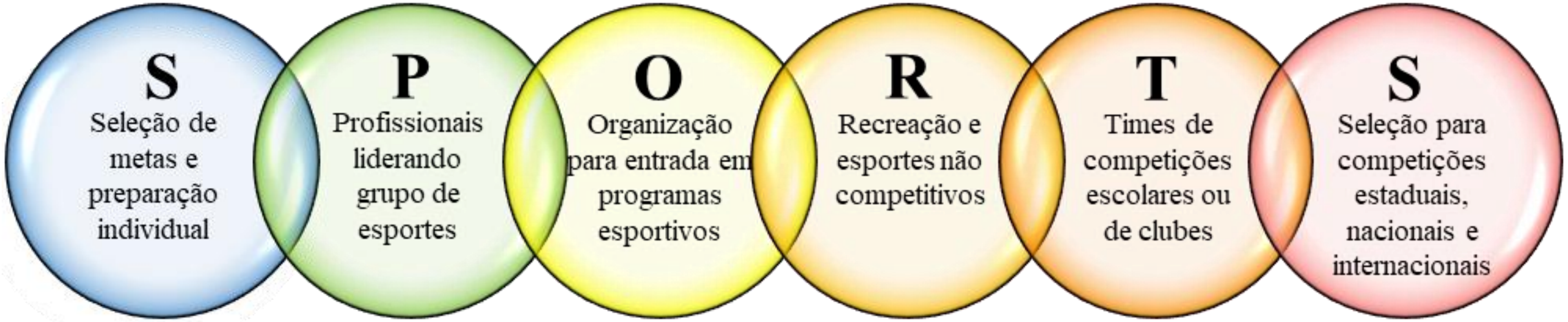

Modelo de Participação SPORTS

para pessoas com deficiência

Children's Motor Control
Research Collaboration

Clutterbuck, G. L., de Sousa Junior, R. R., Leite, H. R., & Johnston, L. M., (2024) *The SPORTS Participation Framework: Illuminating the pathway for people with disability to enter into, participate in, and excel at sport.* Brazilian Journal of Physical Therapy

The SPORTS Participation Framework
for people with disabilities

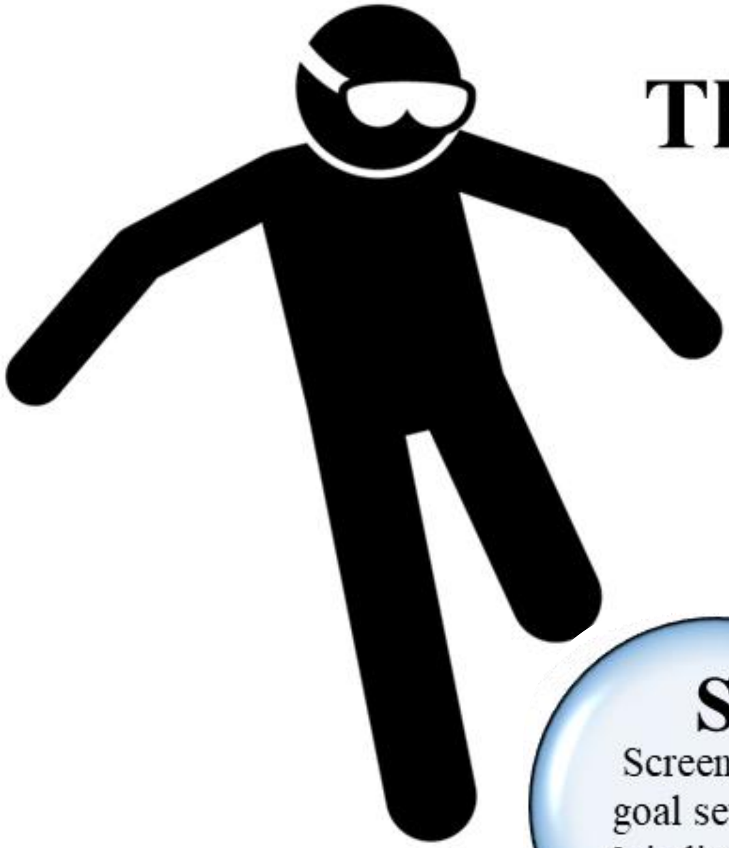

Clutterbuck, G. L., de Sousa Junior, R. R., Leite, H. R., & Johnston, L. M., (2024) *The SPORTS Participation Framework: Illuminating the pathway for people with disability to enter into, participate in, and excel at sport.* Brazilian Journal of Physical Therapy

Children's Motor Control
Research Collaboration

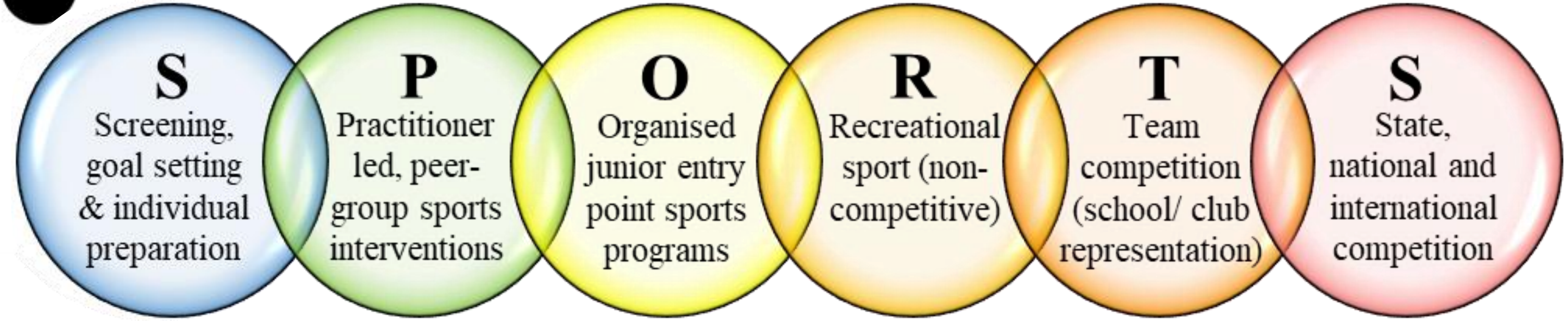

Modelo de Participação SPORTS
para pessoas com deficiência

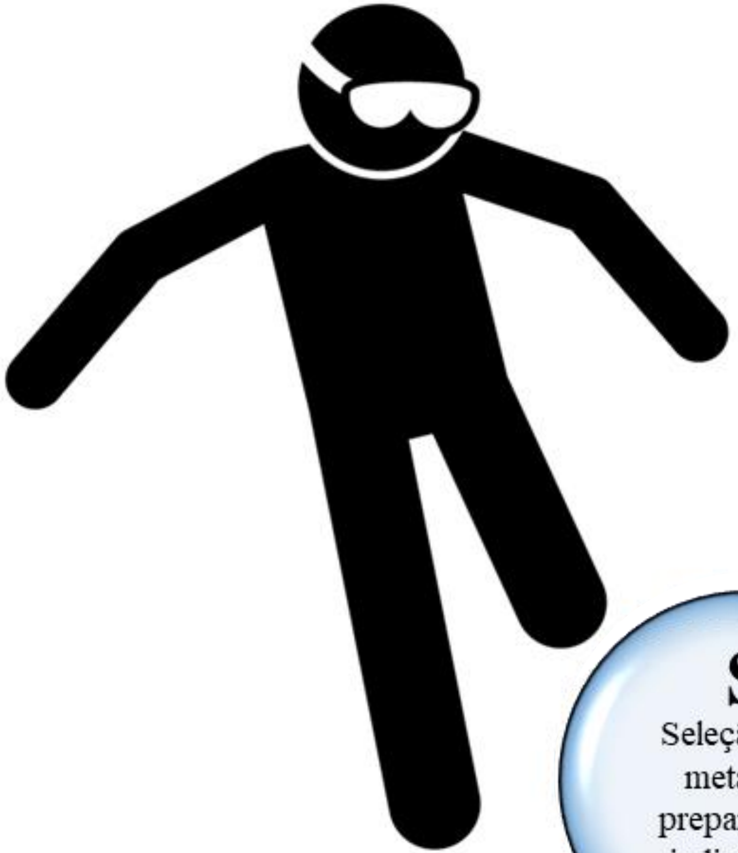

Clutterbuck, G. L., de Sousa Junior, R. R., Leite, H. R., & Johnston, L. M., (2024) *The SPORTS Participation Framework: Illuminating the pathway for people with disability to enter into, participate in, and excel at sport.* Brazilian Journal of Physical Therapy

Children's Motor Control
Research Collaboration

S Seleção de metas e preparação individual

P Profissionais liderando grupo de esportes

O Organização para entrada em programas esportivos

R Recreação e esportes não competitivos

T Times de competições escolares ou de clubes

S Seleção para competições estaduais, nacionais e internacionais

Children's Motor Control
Research Collaboration

The SPORTS Participation Framework
for people with disabilities

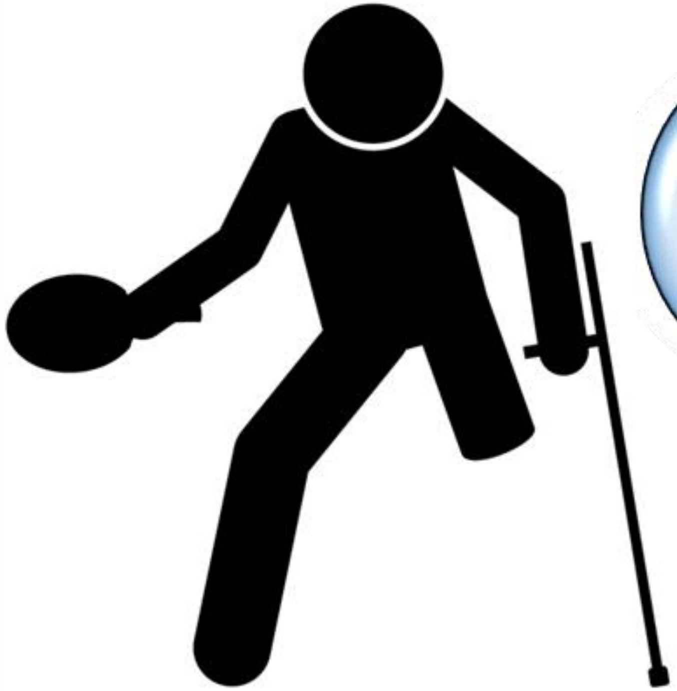

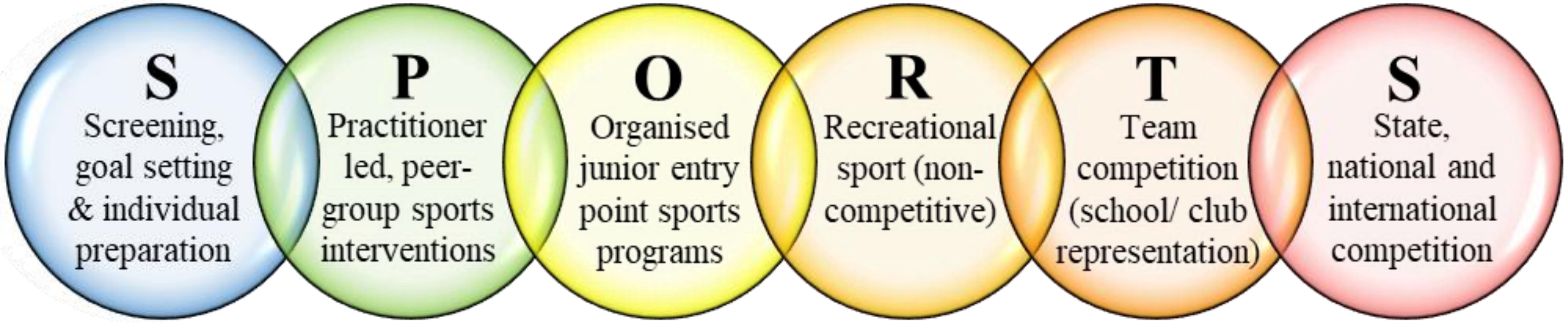

Clutterbuck, G. L., de Sousa Junior, R. R., Leite, H. R., & Johnston, L. M., (2024) *The SPORTS Participation Framework: Illuminating the pathway for people with disability to enter into, participate in, and excel at sport.* Brazilian Journal of Physical Therapy

Children's Motor Control
Research Collaboration

O Quadro de Participação Esportiva *The SPORTS Participation Framework* para pessoas com deficiência

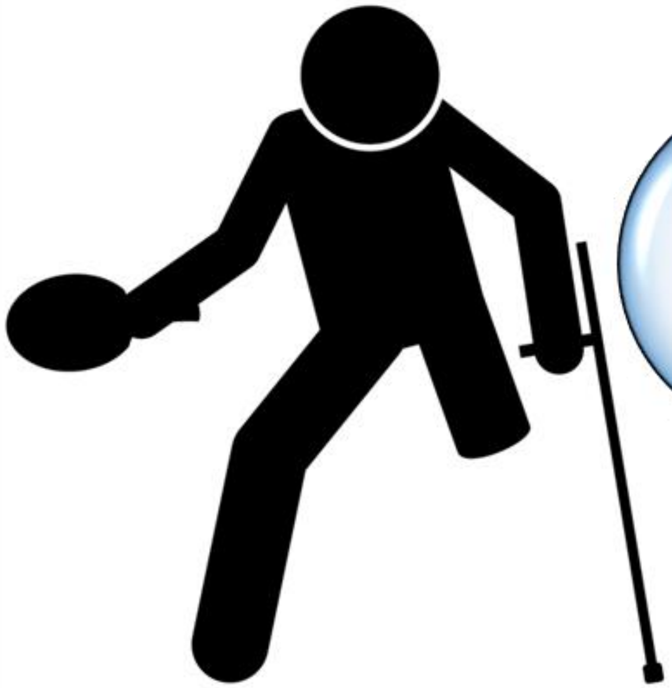

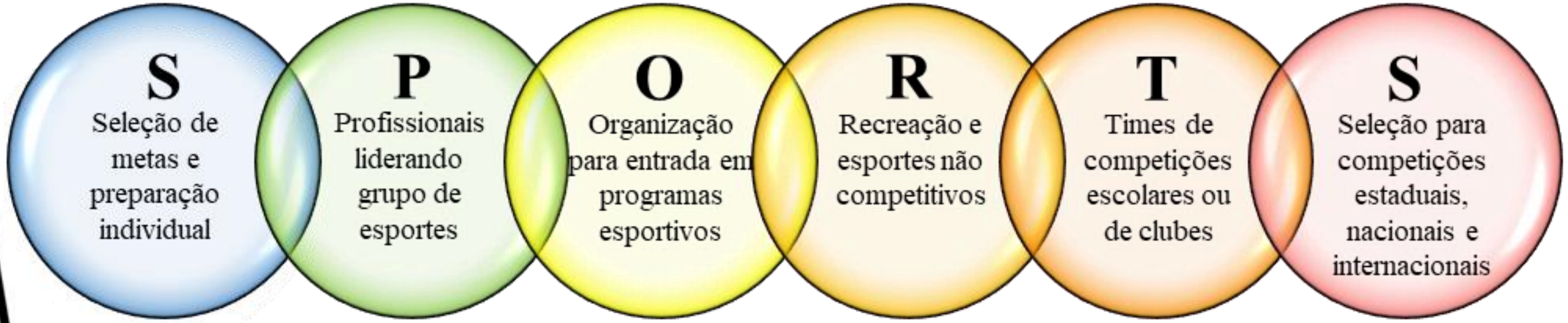

Clutterbuck, G. L., de Sousa Junior, R. R., Leite, H. R., & Johnston, L. M., (2024) *The SPORTS Participation Framework: Illuminating the pathway for people with disability to enter into, participate in, and excel at sport.* Brazilian Journal of Physical Therapy

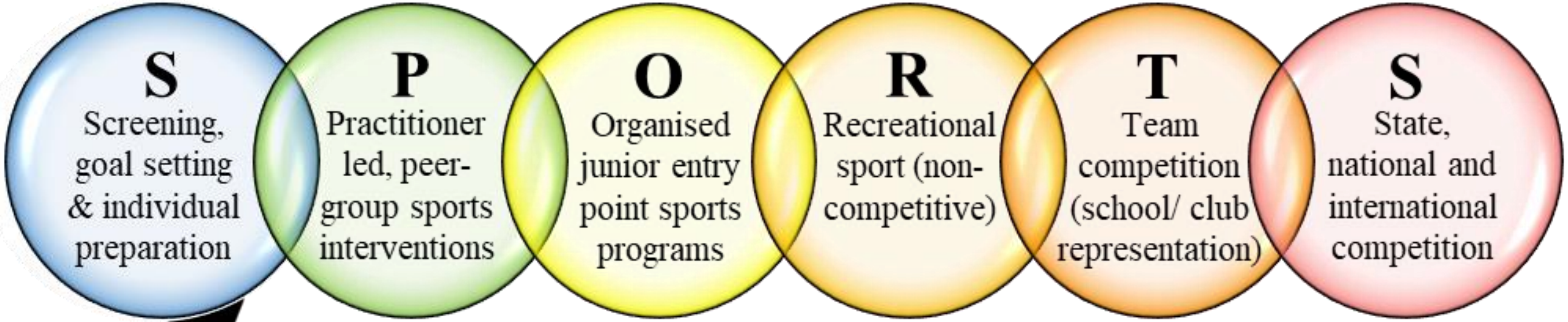

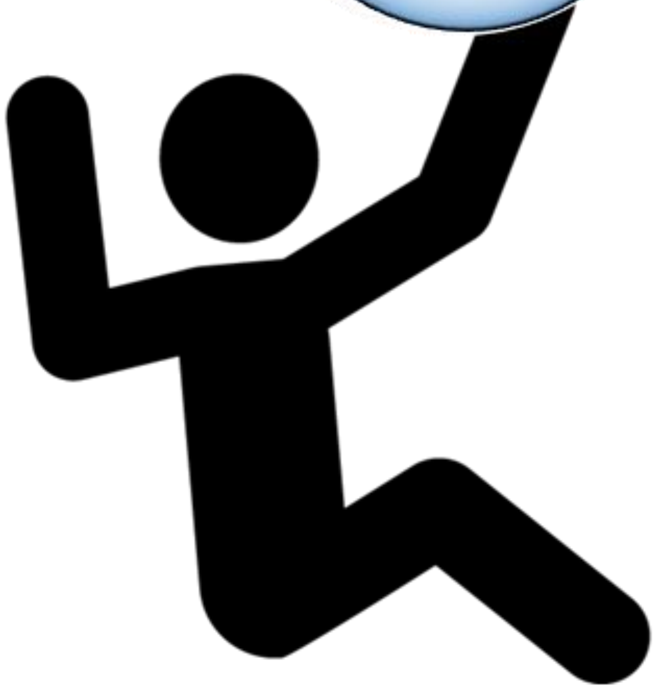

The SPORTS Participation Framework

for people with disabilities

Clutterbuck, G. L., de Sousa Junior, R. R., Leite, H. R., & Johnston, L. M., (2024) *The SPORTS Participation Framework: Illuminating the pathway for people with disability to enter into, participate in, and excel at sport.* Brazilian Journal of Physical Therapy

Children's Motor Control
Research Collaboration

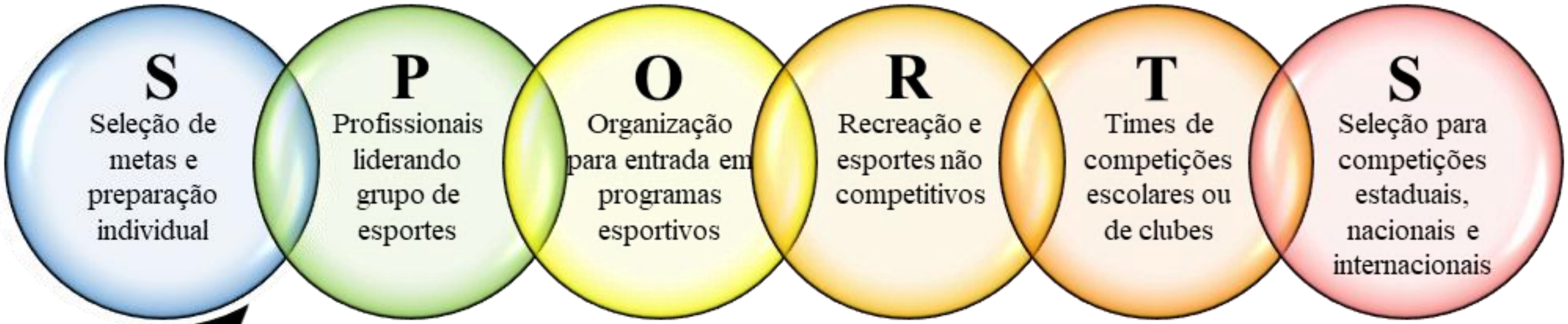

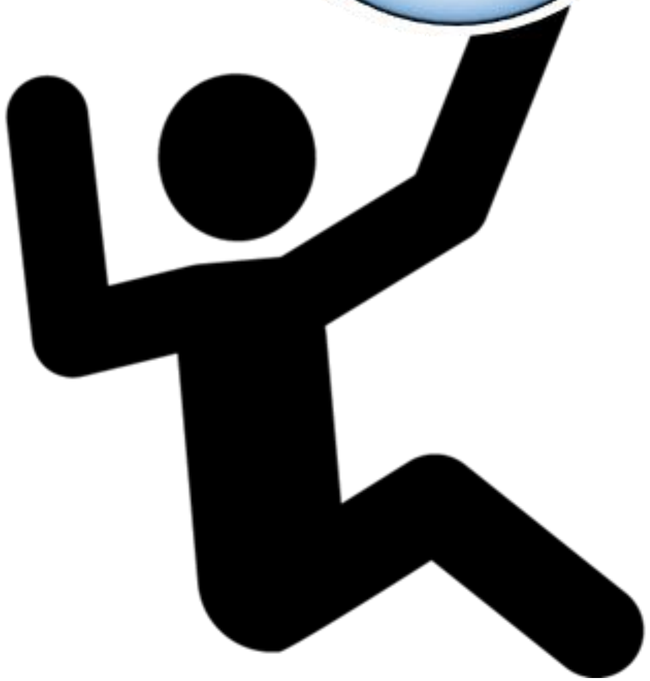

Modelo de Participação SPORTS

para pessoas com deficiência

Clutterbuck, G. L., de Sousa Junior, R. R., Leite, H. R., & Johnston, L. M., (2024) *The SPORTS Participation Framework: Illuminating the pathway for people with disability to enter into, participate in, and excel at sport.* Brazilian Journal of Physical Therapy

Children's Motor Control
Research Collaboration

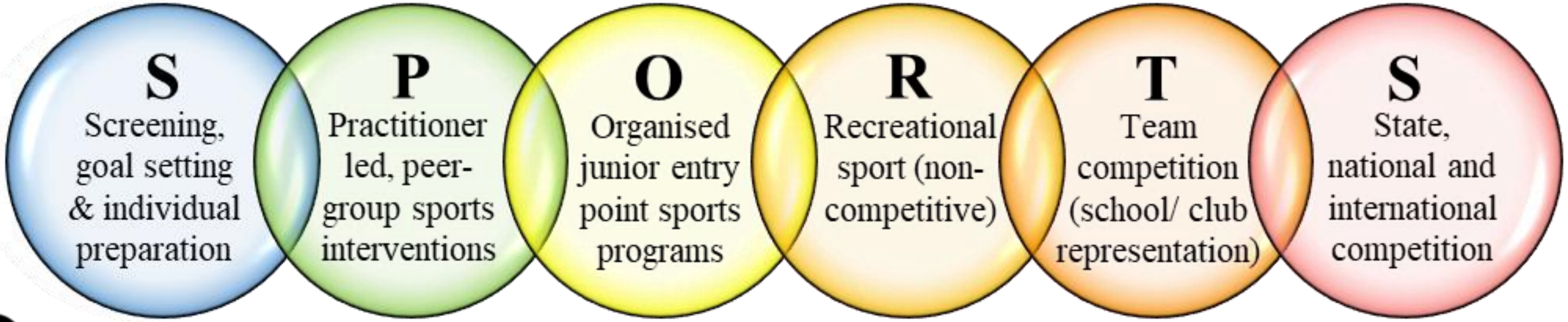

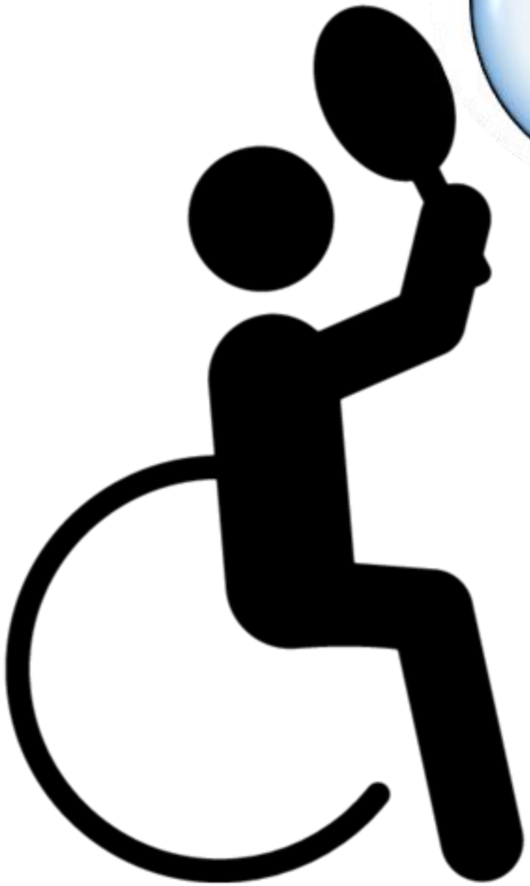

The SPORTS Participation Framework

for people with disabilities

Children's Motor Control
Research Collaboration

Clutterbuck, G. L., de Sousa Junior, R. R., Leite, H. R., & Johnston, L. M., (2024) *The SPORTS Participation Framework: Illuminating the pathway for people with disability to enter into, participate in, and excel at sport.* Brazilian Journal of Physical Therapy

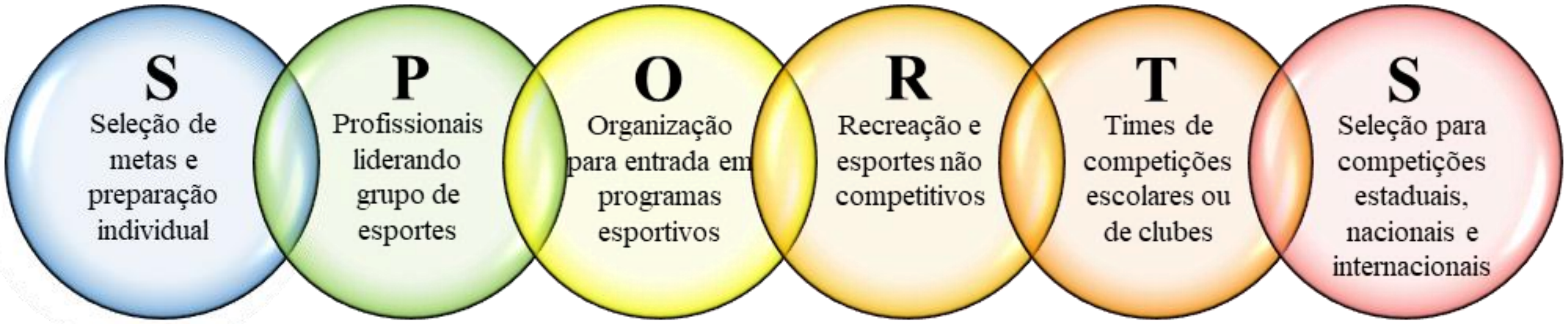

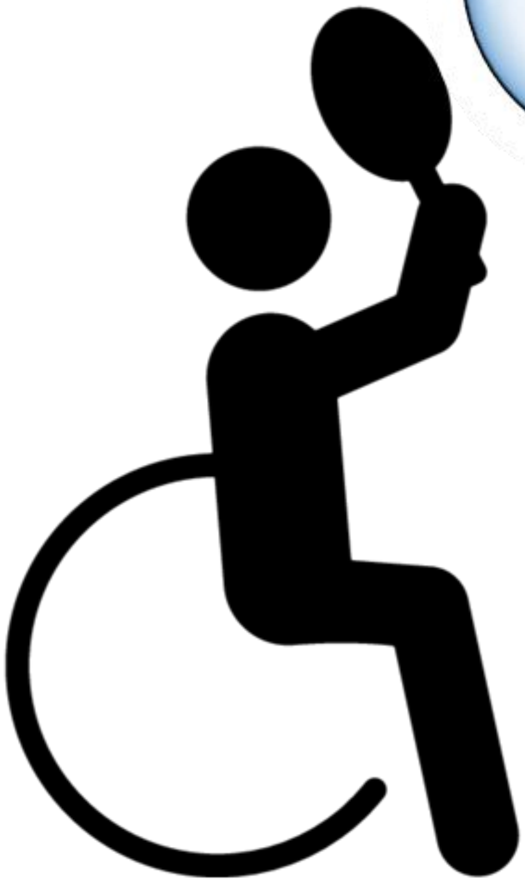

Modelo de Participação SPORTS

para pessoas com deficiência

Children's Motor Control Research Collaboration

Clutterbuck, G. L., de Sousa Junior, R. R., Leite, H. R., & Johnston, L. M., (2024) *The SPORTS Participation Framework: Illuminating the pathway for people with disability to enter into, participate in, and excel at sport.* Brazilian Journal of Physical Therapy

The SPORTS Participation Framework

for people with disabilities

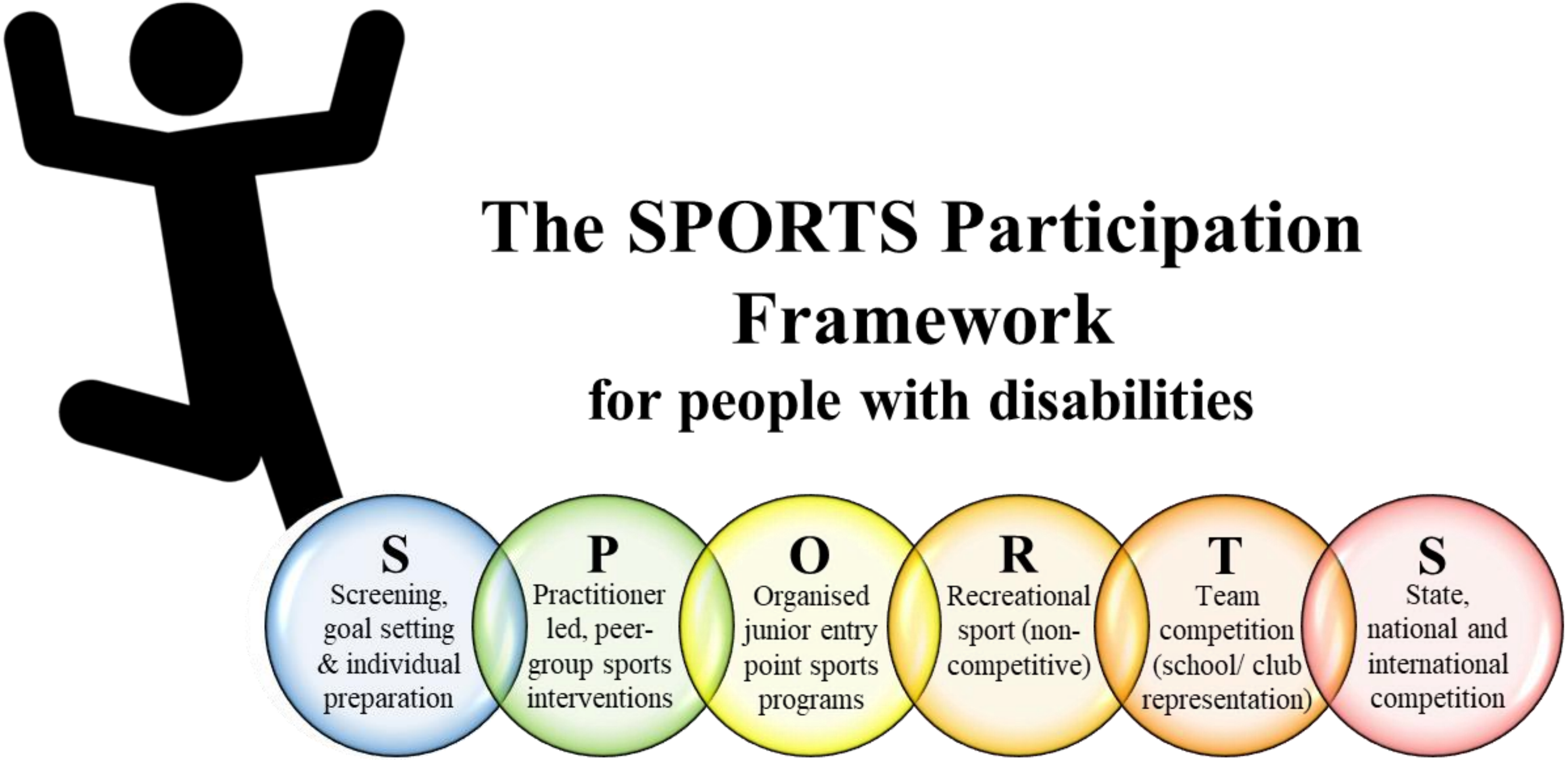

Clutterbuck, G. L., de Sousa Junior, R. R., Leite, H. R., & Johnston, L. M., (2024) *The SPORTS Participation Framework: Illuminating the pathway for people with disability to enter into, participate in, and excel at sport.* Brazilian Journal of Physical Therapy

Children's Motor Control
Research Collaboration

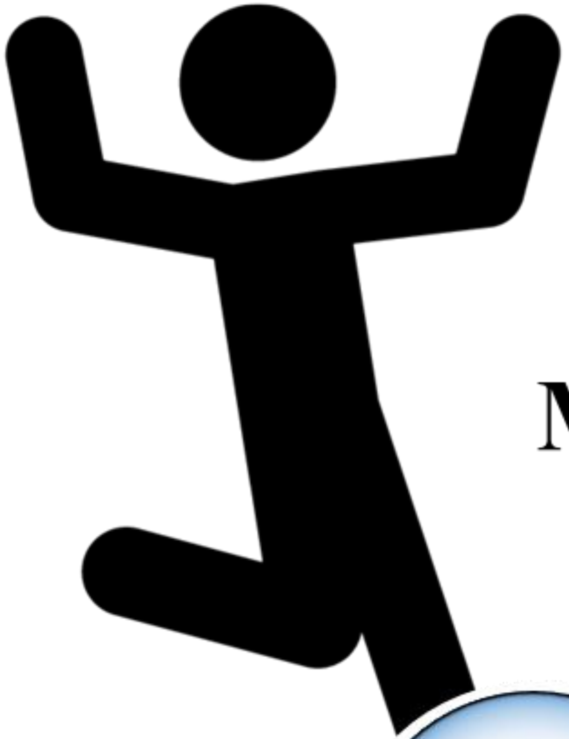

Modelo de Participação SPORTS
para pessoas com deficiência

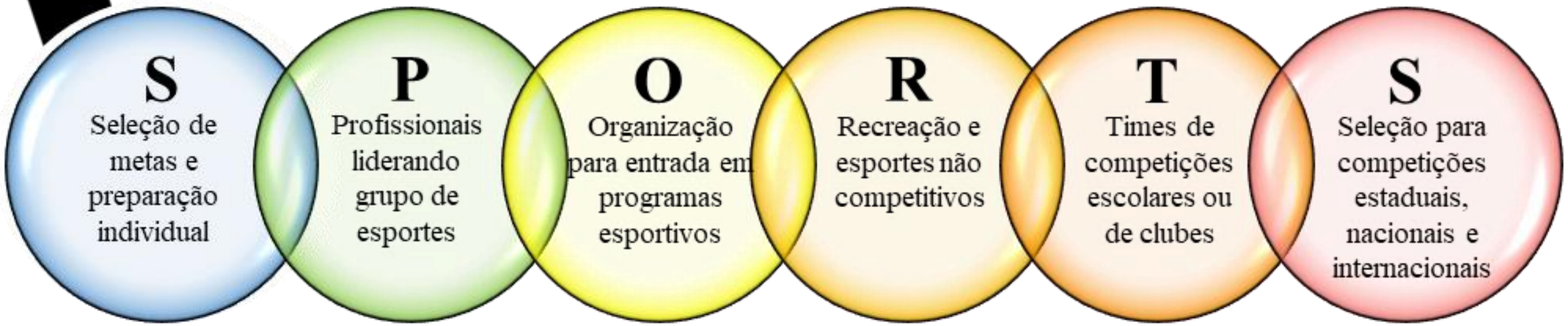

Clutterbuck, G. L., de Sousa Junior, R. R., Leite, H. R., & Johnston, L. M., (2024) *The SPORTS Participation Framework: Illuminating the pathway for people with disability to enter into, participate in, and excel at sport.* Brazilian Journal of Physical Therapy

Children's Motor Control
Research Collaboration

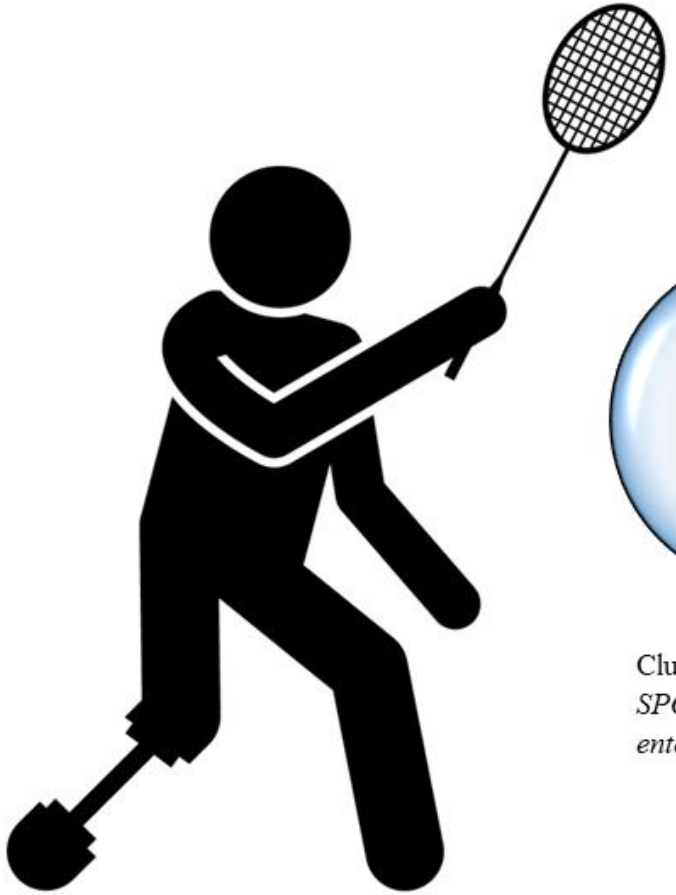

The SPORTS Participation Framework
for people with disabilities

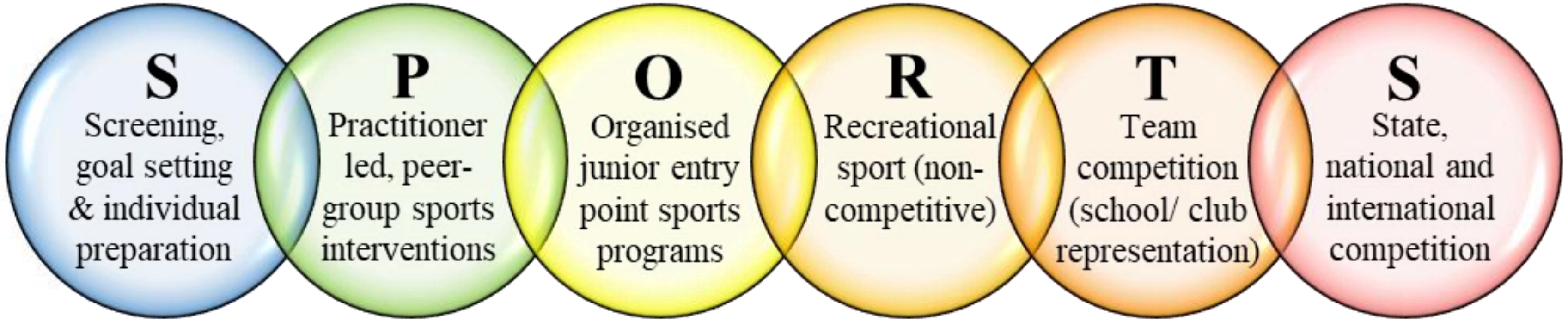

Clutterbuck, G. L., de Sousa Junior, R. R., Leite, H. R., & Johnston, L. M., (2024) *The SPORTS Participation Framework: Illuminating the pathway for people with disability to enter into, participate in, and excel at sport.* Brazilian Journal of Physical Therapy

Children's Motor Control
Research Collaboration

Modelo de Participação SPORTS

para pessoas com deficiência

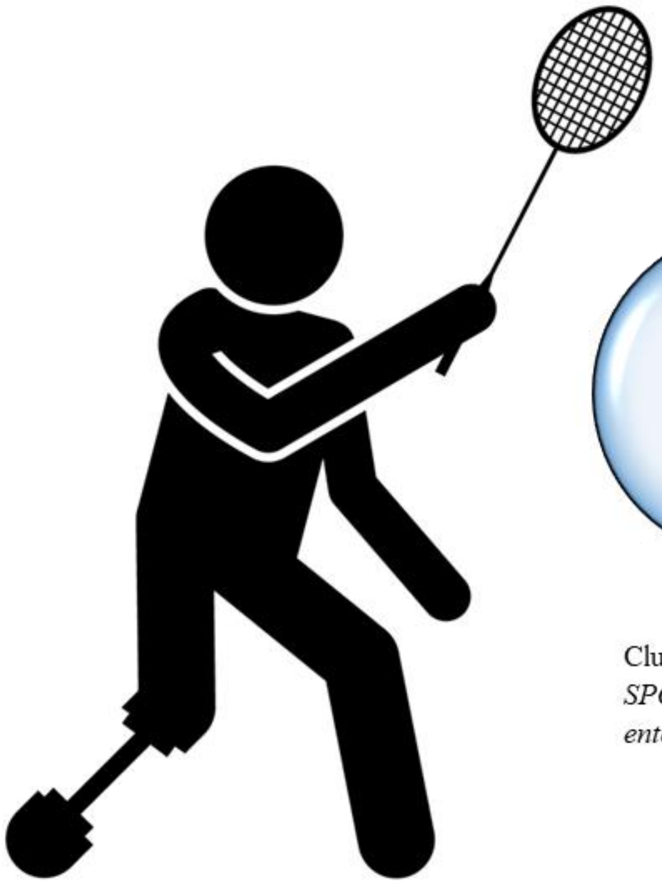

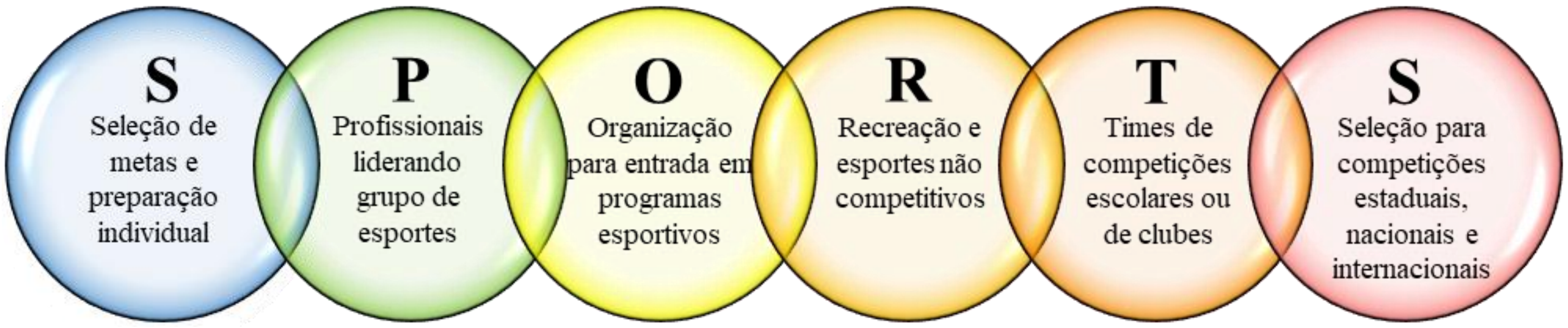

Clutterbuck, G. L., de Sousa Junior, R. R., Leite, H. R., & Johnston, L. M., (2024) *The SPORTS Participation Framework: Illuminating the pathway for people with disability to enter into, participate in, and excel at sport.* Brazilian Journal of Physical Therapy

Children's Motor Control
Research Collaboration
