# Supplementary material for: The SPORTS Participation Framework: illuminating the pathway for people with disability to enter into, participate in, and excel at sport
Source: Braz J Phys Ther. 2024 May 22;28(3):101081. doi: 10.1016/j.bjpt.2024.101081 (PMC11208908; doi:10.1016/j.bjpt.2024.101081)
Supplement: Supplementary file 2 [file mmc2.docx]

Appendix

| **Case study 1**: Meet Ali  Ali is 16 years old and has a diagnosis of spastic quadriplegic cerebral palsy, GMFCS IV.  Ali’s non-linear SPORTS pathway is typical of someone with high-support needs. They began engaging in sport through an aquatic physical therapy program targeting body structure and function level impairments when they were 14. Ali’s physical therapist encouraged their interest in learning to swim during these sessions. They began attending a community pool with their father on the weekends to practice swimming. Ali then joined a research program investigating performance-focused swimming training for people with high support needs, where they progressed directly to state-level competition.  Unlike most people, the majority of Ali’s sports journey has been facilitated at the final ‘S’ stage of State, National, and International competition. Ali was unable to transition smoothly between the stages of the SPORTS Participation framework due to the absence of ‘P’, ‘O’, and ‘T’ stage activities for someone with their level of support needs. Without access to the research program, it is unlikely that Ali would have progressed into performance focused sport. It is also likely that without direct sports-specific training, changes to Ali’s circumstances, progression of physical impairments, and/or changes to motivation would have resulted in them dropping out of recreational sport participation.  Figure 1. Alis SPORTS Stage Journey: |
| --- |

| *Activities Ali participated in during their SPORTS Pathway* | | | | | |
| --- | --- | --- | --- | --- | --- |
| S | P | O | R | T | S |
| Screening, goal setting,  & individual preparation | **Practitioner-led,  peer-group sports intervention** | **Organised junior  entry-point program** | **Recreational sport** | **Team competition** | **State,  national, & international competition** |
| Ali received sports-specific screening, goal-setting, and individual preparation only after engaging with the high-performance research team. This included assessment and intervention relating to range of motion, strength, cardiorespiratory function, and swimming specific skills. Goal setting focused on swimming speed and participation in elite competition | *No ‘P’ stage interventions were available for Ali’s ability, in their geographical area, or for their sport of interest (swimming)* | *No ‘O’ stage interventions were available for Ali’s ability, in their geographical area, or for their sport of interest (swimming)* | Ali participated in swimming activities with the support of their father in a community pool.  *Ali was not able to access any group recreational activities as they were not available for Ali’s ability, in his geographical area, or for his sport of interest (swimming)* | *No ‘T’ stage interventions were available for Ali’s ability, in their geographical area, or for their sport of interest (swimming)* | Ali has participated in performance focused training and state competition, facilitated by a research program. |

**Case study 2:** Meet Kate

Kate is 8 years old and has a diagnosis of autism spectrum disorder (ASD). Her goal was to start participating in the recreational physical activity program at her school.

Kate began her SPORTS pathway in ‘S’ stage screening, goal setting, and individual preparation. During this time, Kate had a very busy week schedule where she accessed twice weekly Physical Therapy, Occupational Therapy, Speech Therapy, and Psychology sessions, with the primary focus on her running performance. Kate’s running skills improved significantly after one semester of physical therapy sessions, however during recreational activities in her school, Kate continued to play alone and avoid running activities. Kate is very shy and insecure when she was around other children and lacked confidence in her running.

Figure 2. Kate’s SPORTS Stage Journey:

Kate’s physical therapist referred her to a ‘P’ stage Practitioner-led sports group in her community. During this 8-week program, Kate trained her running skills together with other five children with ASD of the same age. Kate was introduced to different sports such as athletics and soccer. Kate’s parents report that while they noticed her physical skills improve, particularly her ball skills, the biggest improvement was in her social skills and confidence. After participating in the ‘P’ stage intervention, Kate was able to achieve her goal of participating in her school recreational physical activity program. She attends this program twice a week and does not regularly attend individual physical therapy anymore.

For Kate, her biggest barrier to achieving her goal was her confidence and social skills, which were best targeted using a ‘P’ stage group intervention. This allowed Kate to transition from the ‘S’ Screening, goal setting, and individual preparation into a recreational program with a short-term, low-cost intervention.

| *Activities Kate participated in during their SPORTS Pathway* | | | | | |
| --- | --- | --- | --- | --- | --- |
| S | P | O | R | T | S |
| Screening, goal setting  & individual preparation | **Practitioner-led,  peer-group sports intervention** | **Organised junior  entry-point program** | **Recreational sport** | **Team competition** | **State,  national, & international competition** |
| Kate received sports-specific screening, goal-setting, and individual preparation to improve her running skills. Kate spent a significant amount of time at this stage, which may have been reduced by identifying that a group intervention may have been able to better target her primary barriers to participation. | Kate participated in a group with similarly aged children with ASD. This addressed barriers to participation across all physical literacy domains. | *Kate’s goal of attending her school recreational program did not require sport-specific training at this stage*. | Kate started to participate in a recreational program at her school | Not applicable | Not applicable |

**Case study 3:** Meet Ryan

Ryan has a diagnosis of spina bifida (L1 level) and wants to compete at the Paralympics in wheelchair basketball.

Ryan is 18 years old and uses a wheelchair for mobility in the community. Ryan started to participate in a community wheelchair basketball team six months ago, and trains twice a week. Ryan is very skilled in basketball and has been invited to join a competitive college team. However, he has been feeling increasing pain in his right shoulder when he trains. Ryan’s coach suggested he seek out physical therapy treatment to treat his injury, reduce pain, improve performance, and reduce his risk of further injury and time off training as his training load increases.

Figure 3. Ryan’s SPORTS Stage Journey

| *Activities Ryan participated in during their SPORTS Pathway* | | | | | |
| --- | --- | --- | --- | --- | --- |
| S | P | O | R | T | S |
| Screening, goal setting,  & individual preparation | **Practitioner-led,  peer-group sports intervention** | **Organised junior  entry-point program** | **Recreational sport** | **Team competition** | **State,  national, & international competition** |
| Ryan was referred to physical therapy sessions by his coach to be able to manage his injury and prevent future injury during the higher training loads expected in competition. | *No ‘P’ stage interventions were available for Ryan’s ability in their geographical area.* | *No ‘O’ stage interventions were available for Ryan’s ability in their geographical area.* | Ryan commenced his SPORTS pathway when he joined a community recreational wheelchair basketball program. | Ryan has been invited to compete in a college wheelchair basketball team, however, is impacted by pain in his shoulder. | Ryan would like to compete at this level in the future. |
